# Structural Causes of Brittleness Changes in Aluminosilicate Glasses with Different Cooling Rates

**DOI:** 10.3390/ma17071595

**Published:** 2024-03-31

**Authors:** Liqiang Zheng, Shimin Liu, Fushun Ji, Lianjie Tong, Shiqing Xu

**Affiliations:** 1 State Key Laboratory of Metastable Materials Science and Technology, Yanshan University, Qinhuangdao 066004, China; zlq202311@163.com (L.Z.); ysutlj@163.com (L.T.); 2 Hebei Building Materials Vocational and Technical College, Qinhuangdao 066004, China; jfsniyjzy@163.com

**Keywords:** MD simulation, aluminosilicate glass, annealing rate, ductile/brittle transition

## Abstract

Numerous sources have already demonstrated that varying annealing rates can result in distinct toughness and brittleness in glass. To determine the underlying mechanisms driving this phenomenon, molecular dynamic (MD) simulations were employed to investigate the microstructure of aluminosilicate glasses under different cooling rates, and then uniaxial stretching was performed on them under controlled conditions. Results indicated that compared with short-range structure, cooling rate has a greater influence on the medium-range structure in glass, and it remarkably affects the volume of voids. Both factors play a crucial role in determining the brittleness of the glass. The former adjusts network connectivity to influence force transmission by manipulating the levels of bridging oxygen (BO) and non-bridging oxygen (NBO), and the latter accomplishes the objective of influencing brittleness by modifying the environmental conditions that affect the changes in BO and NBO content. The variation in the void environment results in differences in the strategies of the changes in BO and NBO content during glass stress. These findings stem from the excellent response of BO and NBO to the characteristic points of stress–strain curves during stretching. This paper holds importance in understanding the reasons behind the effect of cooling rates on glass brittleness and in enhancing our understanding of the ductile/brittle transition (DTB) in glass.

## 1. Introduction

Sodium–aluminum–silicate glass plays a crucial role in various technological fields such as medicine, electronics, and telecommunications [1,2]. However, the inherent brittleness of glass products poses a substantial challenge, because it makes them prone to catastrophic fractures when subjected to impacts, scratches, or vibrations. This challenge not only affects the lifespan and quality of the products but also limits their application in more demanding scenarios [3,4]. Consequently, enhancing the toughness and reducing the brittleness of glass has become a primary research focus in the glass industry in recent years.

As a crucial production process in glass shaping, the relaxation of glass structure during annealing has been a focal point of research for scholars. The density, refractive index, hardness, crack resistance, and toughness of glass substantially depend on the thermal history during annealing, and the annealing rate is an important key parameter [5,6,7,8]. Numerous experiments have demonstrated that reducing the cooling rate leads to increased glass hardness and enhanced brittleness [9,10,11,12,13]. Conversely, increasing the cooling rate results in decreased glass brittleness. An evident phenomenon is manifested when glass is subjected to the same load from a Vickers indenter, and a higher cooling rate results in shorter radial crack lengths. Reduced brittleness increases the ability of the glass to resist crack extension [14,15]. Given these phenomena, the difficulty in detecting the non-crystalline structure of glass has hindered direct observation of the microstructural changes involved. However, with advancements in molecular dynamics (MD) techniques, the use of MD simulations to investigate these structural changes has become an indispensable approach. Numerous studies have been conducted to determine the influence of cooling rates on glass structure [16,17,18,19,20], as well as the structural changes during the tensile fracture of glasses with different compositions [21,22,23,24,25], yielding compelling results. However, many unresolved aspects regarding the causes and mechanisms of brittleness changes in glass under different cooling rates are still unresolved. For instance, which specific structural characteristics of glass primarily influence its brittleness? How does the cooling rate control these structural features, thereby affecting the mechanical properties of glass?

In this paper, we first investigated the effect of the cooling rate on the glass microstructure (e.g., radial distribution function, angular distribution, BO/NBO, volume and enthalpy); second, the bond lengths and BO/NBO variations during stretching of different cooled samples were investigated, and it was found that the content of BO/NBO has an important effect on the brittleness due to the location of the sudden change in the content during stretching. Finally, synthesizing the results of the above studies, we compiled the reasons for the different levels of glass brittleness caused by different cooling rates, while rationalizing the important role of voids in glass brittleness. The obtained results explain the varying brittleness of glass with respect to different cooling rates and offer valuable insights for further investigations into the brittle/ductile transition of glass and for the manufacture of glass with better mechanical properties.

## 2. Simulation Details

### 2.1. Glass Preparation

The glass composition under investigation in our study is 25Na_2_O-5Al_2_O_3_-70SiO_2_ (mol. %). This composition holds remarkable commercial and research value in the field of glass manufacturing and aluminosilicate glass research. The glass samples were prepared using the conventional melt-quenching method, and all MD simulations were performed using the two-body potential (SHIK). The long-range Coulomb interactions were treated using the Wolf truncation method, with the inclusion of a short-range repulsive term to prevent particles from fusing together in an unphysical manner (the form and parameters of the potential function are given in the first part of the Supplementary Materials). Cutoff distances of 8 and 10 Å were employed for short-range and Coulombic interactions, respectively [26]. The potential function has been demonstrated to describe the structure and mechanical properties of aluminosilicate glasses reliably and in good correspondence with experimental results [27,28]. All simulations were performed using LAMMPS software (version 2 Aug 2023) [29] with a time step of 1.6 fs and a Nosé–Hoover thermostat and barostat [30,31,32]. A 3D box (size 81.4 Å × 81.4 Å × 81.4 Å, and density 2.45 g/cm^3^) with about 40,000 atoms (assigned by molar composition) with random coordinates was formed and used as the initial structure. It was then equilibrated in an NVT system (constant volume and constant temperature) for 300 ps. Subsequent equilibration of 300 ps at the same temperature and zero pressure in an NPT system (constant atomic number, pressure, and temperature) ensured the memory of the initial configuration was completely lost. The high-temperature melt under 3000 K was then continuously quenched in the NPT system at cooling rates of 0.25, 1, 5, and 30 K/ps to 300 K. This process was followed by a relaxation of 160 ps in the NPT system to relax any internal pressures in the structure. The room temperature was much lower than the glass transition temperature. Longer relaxation did not need to be considered, because the atomic configuration was practically frozen. To obtain statistically significant results, three independent samples for each cooling rate were prepared for later structural analysis.

### 2.2. Fracture Simulations

**Figure 1:**
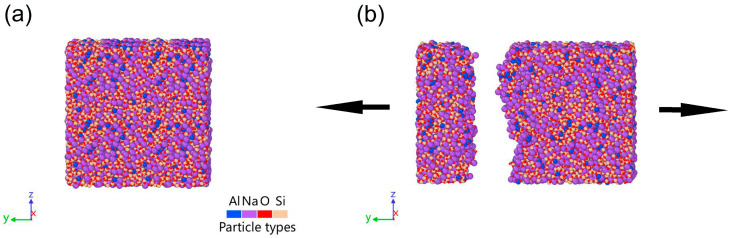
(**a**) Glass model of 0.25 K/ps. (**b**) The direction of stretching of the model and modeled shape at fracture.

The uniaxial tensile fracture simulation was performed by stretching the box in the *y*-direction at 300 K, and the box size was fixed in the other directions. This causes the model to strain only in the y-direction and remain fixed in the other directions. Figure 1a shows the glass model of 0.25 K/ps, and Figure 1b illustrates the direction of stretching of the model. Other rates of glass models and stretching are similar to this one. The strain rate is fixed at 5 × 10^8^/s and uses periodic boundary conditions for all fracture simulations. The NPT ensemble was used during the simulations. The temperature and pressure damping parameters were 0.16 and 1.6 ps, respectively. The stress–strain curve of the tensile process was plotted by integrating the total stress experienced by all atoms in the y-direction under varying strains [33]. The total fracture energy G_F_ was obtained by integrating the stress–strain curve. Similarly, the estimated elastic energy G_E_ was calculated by integrating the stress–strain curve before the peak stress was achieved [24], and the plasticity of G_P_ was determined by calculating the difference between G_F_ and G_E_. Then, the brittleness index B was defined as G_E_/G_F_, reflecting the relative contribution of elastic and plastic deformation of the material during fracture. The analysis of fracture process outcomes was obtained from one quenched sample at each cooling rate. Due to the sufficiently large system size considered in the study, fluctuations between samples can be disregarded as long as the samples did not initiate fracture.

### 2.3. Enthalpy and Void Volume

To ensure that our samples with different cooling rates were obtained in our simulations, the enthalpy of the intrinsic conformation during glass cooling at different temperatures was calculated by Lammps, and the results were output at 1.6 ps intervals from the cooling.

The void volume of each sample and the volume of the whole box were calculated using the Multiwfn code [34]. It attempted to determine whether a lattice (a model divided into an infinite number of square lattices of equal volume) is occupied within the van der Waals radius of any of the surrounding atoms. Moreover, it set the sum of the unoccupied lattices as the void volume of the system. In this paper, we have investigated the van der Waals radii of all the elements and finalized the lattice point spacing to be 0.25 Å, which ensures that the results of the void volume calculations are as accurate as possible while ensuring the speed of the calculations. This method of calculation is consistent with the principle of void volume calculation in other oxide glasses [35,36,37]. In addition, the difference between the box volume and the void volume was named as the structural volume.

### 2.4. Structural Characterization

The pair distribution function (PDF) was used to capture the short-range structure around the cation. Here, *g*(*r*) is defined as $g\left(r\right)=\frac{1}{N\rho }<\sum_{i}^{N}\sum_{j\neq i}^{N}\delta \left(r-r_{ij}\right)>$, where *N* is the number of atoms, *ρ* is the number density, and *δ* is the Dirac function. As r increases, *g*(*r*) oscillates and approaches 1. The bond lengths of Si-O, Al-O, and Na-O were obtained by the transverse coordinate of the highest point of the first peak of the PDF [21,38].

The medium-range structure (BO/NBO) of the network was assessed by computing the coordination numbers of Si, Al, O, and Na atoms. This computation was achieved by enumerating for each atom the number of neighbors within its first coordination shell, with a cutoff chosen as the first minimum after the first peak of the partial PDF. The analysis was used to discriminate BO and NBO, which exhibit either two or one Si/Al atoms in the O first coordination shell, respectively, and NBO is just a bond between oxygen atoms and sodium atoms [39]. In the BO and NBO analysis of the stretching, due to the large fluctuation in the data, the curves were binomially smoothed with the Savitzky–Golay method, and as many curve features as possible were retained, which facilitated subsequent analysis.

## 3. Results and Discussion

### 3.1. Features of Samples with Different Cooling Rates

**Figure 2:**
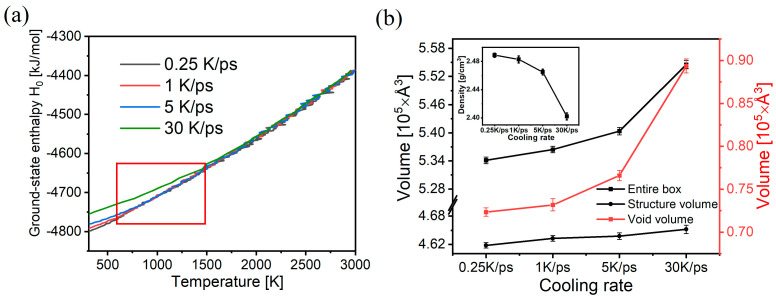
(**a**) Enthalpy changes in initial glass samples upon cooling at different cooling rates (the red region shows the approximate area where the glass transition occurs). (**b**) Box volume, void volume, and structural volume of the final samples obtained at different cooling rates; the inset shows the density of the samples at different cooling rates.

We first focused on the temperature dependence of enthalpy to ensure our simulations yielded samples with different cooling rates. As shown in Figure 2a, the enthalpy of the simulated samples decreases monotonically with decreasing temperature in general, and the slope changes abruptly after a certain temperature is reached (small fluctuations in temperature are easily observed in the high-temperature section for low cooling rates due to the longer observation time, but this does not affect our study of the general trend). This indicates that the system reaches the glass transition region (Tg) and undergoes vigorous relaxation to form an amorphous glass. Below the glass transition region, the slope decreases, because the glass structure is essentially frozen, but the structure still undergoes slow relaxation at this temperature, and this agrees with the experimental phenomena reported previously [40,41]. In addition, the value of Tg can be obtained by linear extrapolation of the high and low temperatures, and the Tg values of the glass at each of the simulated cooling rates were calculated according to Figure 2: 0.25 K/ps for 740 K, 1 K/ps for 780 K, 5 K/ps for 960 K, and 30 K/ps for 1060 K, which is in the range of 730–1060 K, close to the experimental Tg value of 780 K for this glass [42]. Despite the huge difference between the simulated and actual cooling rates, the results are still within the error range. Figure 2a also shows that the Tg region decreases as the cooling rate of the glass decreases, and the final enthalpy of the model also decreases as the cooling rate decreases when room temperature is reached. This result agrees with what has been previously studied: slower cooling rates provide the structure with more time to transition to the equilibrium state and generate more stable structures with lower energies [20]. This phenomenon is also consistent with the concept of the “virtual temperature” of glass [43].

After obtaining the samples with different cooling rates, the void volume, the box volume, and the structure volume of the samples were calculated, as shown in Figure 2b. The void volume changes more evidently with the increase in the cooling rate, where the extreme difference of the void volume is 0.170 × 10^5^ Å^3^, whereas the extreme difference of the structural volume is 0.034 × 10^5^ Å^3^. Therefore, the main reason for the rate-induced changes in the density of the samples is that the different sizes of the void volume are generated. The volume change caused by the structural change is also an important factor.

**Figure 3:**
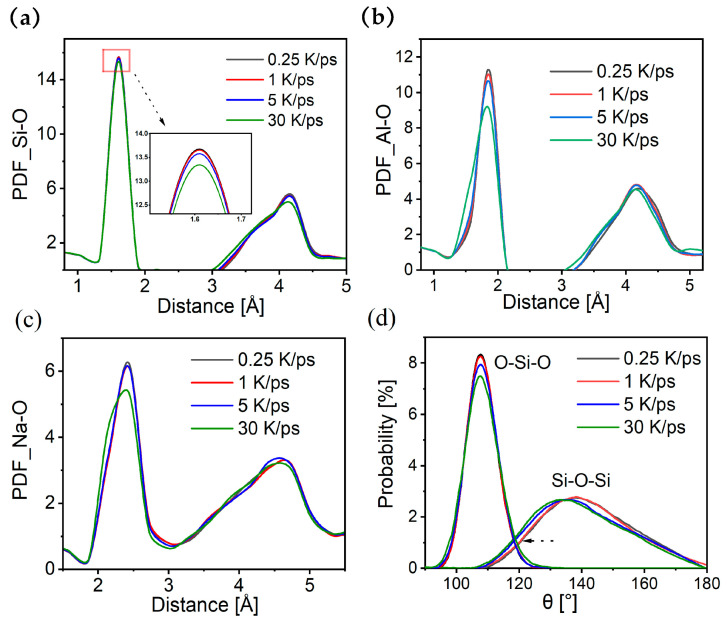
PDFs of samples with different cooling rates: (**a**) Si-O; (**b**) Al-O; (**c**) Na-O. (**d**) The angular distribution of Si-O-Si and O-Si-O.

**Figure 4:**
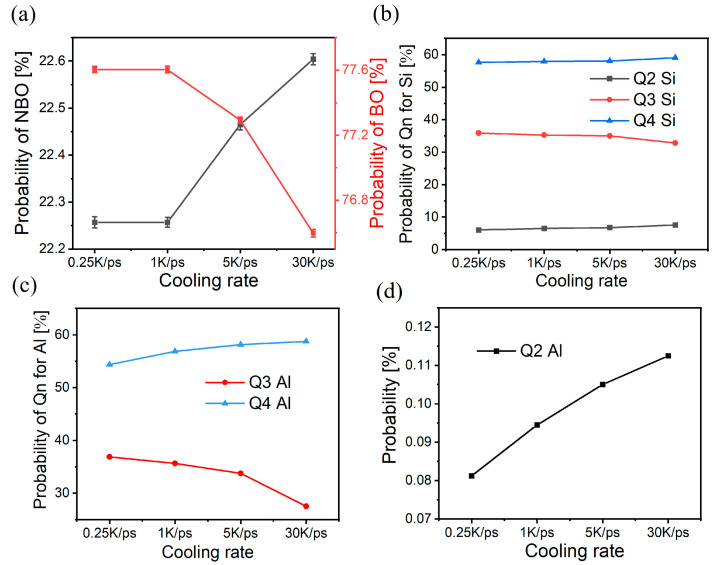
Oxygen states of samples with different cooling rates: (**a**) BO and NBO distributions; (**b**) different forms of Si tetrahedral bridged oxygen distributions; (**c**) Al tetrahedral bridging oxygen distributions for Q4 and Q3; (**d**) Al tetrahedral bridging oxygen distribution for Q2.

Figure 3a–c show the Si-O, Al-O, and Na-O PDF for samples with different cooling rates, respectively. The PDF trends are essentially the same at all rates, the peak positions are consistent with previous synchrotron radiation tests [44], and there are no significant shifts in peak positions. However, it is worth noting that the heights of the first and second peaks of each PDF decrease with increasing cooling rates, which often represents a decrease in the orderliness of the glass network. In addition, the first peaks of Al-O and Na-O not only decreased in height at the rate of 30 K/ps but also became fatter in the left half of the peaks, which might be related to the increase in NBO and the increase in Q4Al (see Figure 4). Figure 3d calculates the angular distributions of Si-O-Si and O-Si-O, and the O-Si-O angle decreases with the peak value of the cooling rate, whereas the Si-O-Si is shifted to a small angle. This finding is consistent with the phenomenon observed by other researchers [45,46]. The phenomenon that only the peak of the O-Si-O angle decreases indicates that the main shape of the Si tetrahedron is not affected by the cooling rate; the high cooling rate reduces the ordering of the glass network, and the decrease in the peak represents the possibility of the elevation of the other angles. The Si-O-Si angular distribution moving to higher angles with a decreasing cooling rate may be a compromise in the connections between the silicon tetrahedra to accommodate the decrease in volume and increase in density.

Figure 4 shows the BO/NBO distribution for samples with different cooling rates. As shown in Figure 4a, the BO content decreases, whereas the NBO content increases with the increase in cooling rate. Figure 4b shows that the contents of Q4Si and Q2Si slightly increase with the increase in cooling rate, whereas the content of Q3Si decreases, and these results are consistent with the simulation results of other scholars [16]. This outcome means the reaction 2Q3Si → Q4Si + Q2Si has occurred. Figure 4c,d show a change in Q4Al, Q3Al, and Q2Al (although the content of Q2Al appears more variable than Q4Al and Q3Al, and this is caused by the different values taken for the vertical coordinates of the pictures, because Q2Al is too low), which again can be explained in a similar way: 2Q3Al → Q4Al + Q2Al. This result suggests that when the elevated cooling rate provides more voids, Si and Al tetrahedra choose Na^+^ to replenish the voids to maintain the charge balance, which is a compromise to the structural freezing under rapid cooling. The change in BO/NBO can also explain the decrease in the first PDF peak and the peak of the O-Si-O angular distribution with high cooling rates, that is, the tetrahedral connections between tetrahedra or with other atoms are changed.

### 3.2. Structural Changes of the Sample during Stretching

**Figure 5:**
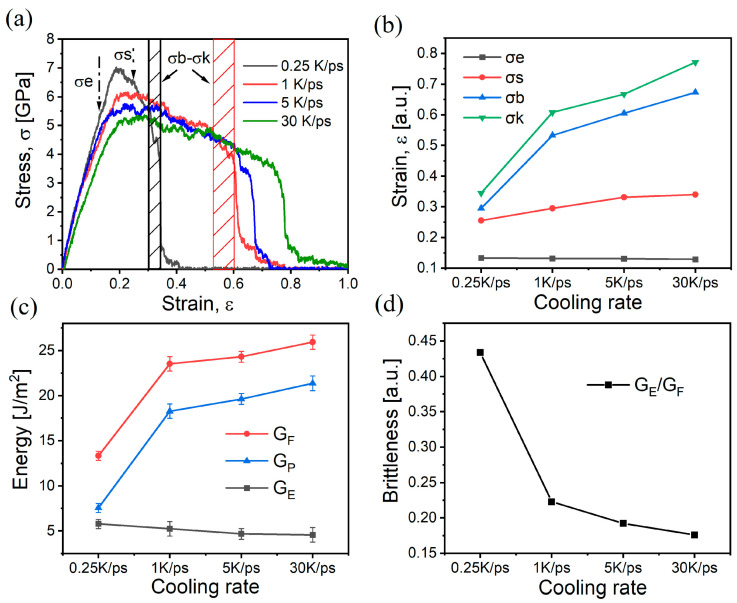
Variation in stress with strain in tension of samples with different cooling rates: (**a**) stress–strain curves, where black-shaded and red-shaded parts are the tensile strength-fracture strength intervals of the samples at the cooling rates of 0.25 K/ps and 1 K/ps, respectively; (**b**) location of mechanical characteristic points in the stress–strain curve for each rate sample; (**c**) fracture energies, elasticity, and plasticity of the samples with different cooling rates obtained from the stress–strain curves; (**d**) brittleness of samples at different cooling rates.

The stress–strain curve of the glass obtained from the simulation is given in Figure 5a. The approximate positions of the elastic limit σe, yield limit σs, tensile strength σb, and fracture strength σk are shown in the curve at 0.25 K/ps in accordance with the analysis method of the stress–strain curve [47]. Feature points for different rates are summarized in Figure 5b. As the cooling rate decreases, the modulus of elasticity of the glass rises (the linear phase at the beginning), and the ductility decreases (i.e., the ability of the material to elongate under tensile loading). This result is in line with previous experimental and simulation studies by scholars showing that low cooling rates elevate glass brittleness [13,14,27]. Furthermore, the modulus of elasticity derived from the simulations in this paper is in the range of 48–60 GPa, which corresponds well with the experimental value of 64 GPa [26], indicating the reliability of the potential function. In Figure 5b, it is worth noting that in addition to σe, all of σs, σb and σk have more obvious changes, which indicates that the ductility changes more and the elasticity changes less with different cooling rates during tensile process. Figure 5c calculates the fracture energy (G_F_), elastic energy (G_E_), and plastic energy (G_P_) of the sample during the tensile process, and the brittleness is sequentially calculated as in Figure 5d. The plastic and fracture energies in Figure 5c change more significantly with different cooling rates compared to the elastic energy, which is consistent with the pattern of the characteristic points in Figure 5b. Therefore, more changes in ductility resulted in more changes in plastic energy and led to an increase in fracture energy, with a consequent eventual reduction in glass brittleness. The change in ductility shows an important role in the brittleness.

**Figure 6:**
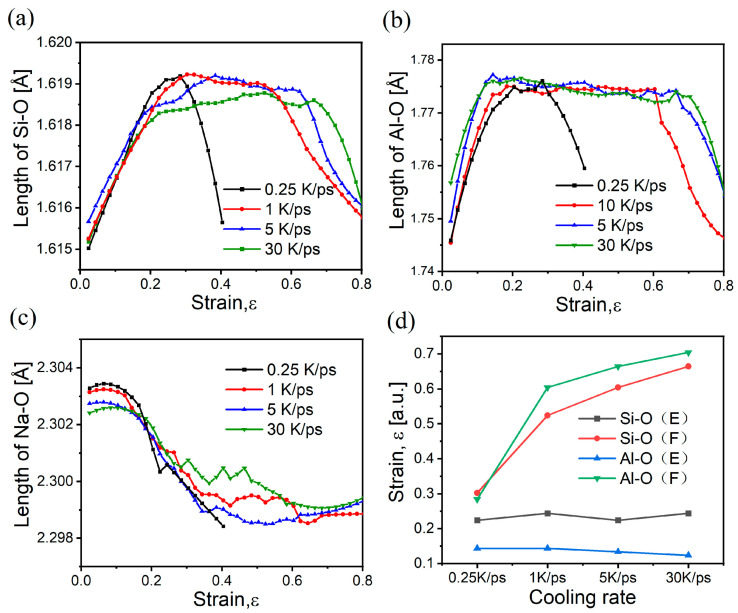
Variation in bond length with strain for samples stretched at different cooling rates; (**a**) Si-O; (**b**) Al-O; (**c**) Na-O; (**d**) the characteristic turning points of the Si-O and Al-O bonds with stretching in the elastic phase (E) and the fracture phase (F).

Bond lengths at different strains were obtained during the tensile process through PDF analysis to investigate the structural changes induced by stretching, as illustrated in Figure 6. Regardless of the cooling rate of the samples, the Si-O bond length and Al-O bond length exhibit substantial changes in the strain range associated with structural transformations, namely, σe-σs and σb-σk, where the former reflects the brittle/ductile transition, and the latter reflects the fracture transition. However, comparing Figure 5b and Figure 6d, this shows that the Si-O bond in the elastic phase reaches stability near σs, with an overall change of three decimal places, whereas the Al-O bond stabilizes near σe, with a change of two decimal places. In addition, the Si-O bond decreases abruptly near σb during the fracture phase, whereas the Al-O bond is near σk. These phenomena indicate that Si-O bonds are more rigid than Al-O bonds, whereas Al-O bonds are more elastic [22,48]. Moreover, the Si-O bond will break preferentially to the Al-O bond when the model is destabilized, with the Al-O bond being more resistant to breakage, which will improve the ductility of the glass. As shown in Figure 6c, the Na-O bond has a small increase in the elastic phase before it starts to decrease continuously near σs. This reflects the property that Na-O bonds are more prone to changing the coordination environment in order to allow the structure to reach charge equilibrium quickly. The continued shortening of the Na-O bond during subsequent stretching is related to the Na compensating for bond breakage during the structural change process, which raises the coordination and becomes more constrained.

**Figure 7:**
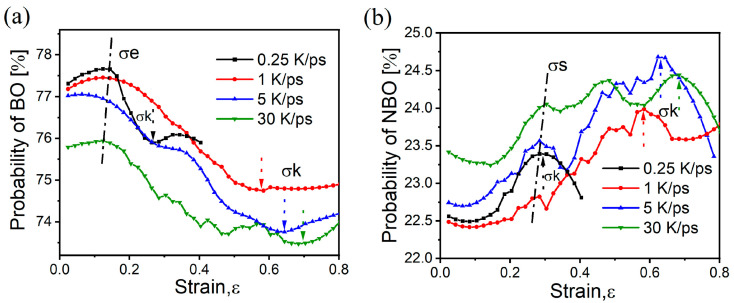
Variation in BO and NBO with strain during stretching of samples at different cooling rates: (**a**) BO; (**b**) NBO; The arrows indicate the position of each feature parameter.

To provide a more comprehensive explanation of the variation in bond lengths during the stretching, the changes in the content of BO and NBO in the glass were analyzed, as illustrated in Figure 7. Regardless of the cooling rate of the samples, the overall content of BO decreases with increasing strain, exhibiting distinct abrupt changes at the characteristic points of σe and σk. Specifically, during the transition from elasticity to plasticity, the BO content initially increases and then decreases, whereas during the extension to fracture and the formation of new surfaces, the BO content initially decreases and then increases. Figure 7b shows the NBO content at the characteristic points has a trend opposite to that of BO, and the overall NBO content increases with increasing strain. The NBO content has an evident bulging change at the yield limit σs, which was not observed in BO. The behavior at the yield point σs indicates that NBO is a critical structural element responsible for the irreversible deformation of the sample, and it is more inclined toward accomplishing irreversible structural rearrangement during the tensile process [21]. By contrast, BO primarily plays a role in maintaining the connectivity of the network structure [23]. This finding is evident in the increase in BO content during the glassy elastic stage to resist strain and the subsequent increase in content to form a new network after fracture.

**Figure 8:**
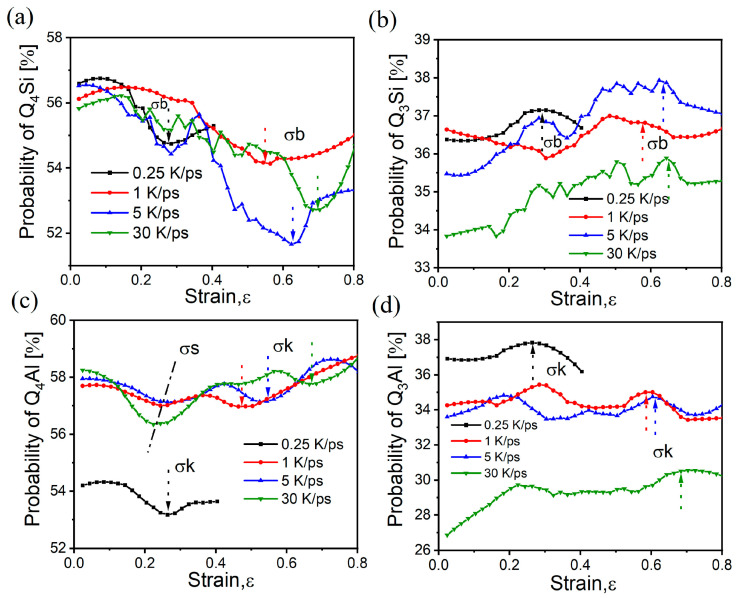
Major structures with strain during stretching of samples with different cooling rates: (**a**) Q4Si; (**b**) Q3Si; (**c**) Q4Al; (**d**) Q3Al; arrows in the figure mark the characteristic locations of the variations.

Figure 8a,b show the variation in Q4Si and Q3Si with strain, respectively, whereas Figure 8c,d show Q4Al and Q3Al, respectively. Although both are BO units and show sensitivity to the characteristic point of the fracture stage, the BO units of Si are all more fluctuating, and the mutation positions are near σb, whereas the BO units of Al are all smoother, and the mutation positions are near σk. These results are consistent with the mutation positions of the bond lengths of both during stretching. Moreover, they all undergo a Q4→Q3 transition (judging by the change in the concavity of the curve) when the curve fluctuates violently near the characteristic point. Combined with the change rule of BO/NBO, it can be judged that the structural reorganization during stretching generates more NBO.

Figure 7 and Figure 8 explain the change in bond lengths in Figure 6. The large amount of BO and NBO content change fully affects the second-nearest neighbor condition of Si-O, Al-O, and Na-O, and the more different second-nearest neighbors cause the corresponding change of bond lengths. For example, the continuous decrease in Na-O bond length with strain can be interpreted as the Na coordination number rises with the NBO and becomes more constrained, resulting in a continuous shortening of the bond length.

### 3.3. Mechanism of Annealing Rate on Brittleness

In our study in Section 3.1, it was found that different cooling rates have an effect on the microstructure of the glass, but more so on the void volume size. In Section 3.2, it was found that the higher the cooling rate, the lower the glass brittleness and the better the ductility; moreover, the mechanical characteristic points related to the brittleness calculations correspond well with the position of the sudden change in the BO/NBO content of the glass during stretching. Therefore, when controlling the position of the sudden change of the BO/NBO content during stretching, the final brittleness of the glass will be controlled. And any factor that can affect the location of the mutation in BO/NBO content is a cause that can affect glass brittleness. This finding can also be corroborated with Zhen Zhang’s discovery of the effect of NBO on the inelastic origin of glass [21] and Tang’s discovery of the effect of rigid clusters on brittleness [22]. And this illustrates the key role of BO/NBO in glass stretching. Therefore, combining the findings of Section 3.1 and Section 3.2, how does the cooling rate affect the position of the sudden change in BO/NBO content during stretching and thus the brittleness of the glass? We think there are two points from the analysis.

A. Influence of short- and medium-range structures (MRO)

In Figure 3 and Figure 4, it is easy to see that different cooling rates result in different glass mid-range structures. Although the change in content is small, the low-cooling-rate samples have more BO and a larger Si-O-Si angle, which also enhances the network connectivity of the glass and makes the network more susceptible to force transfer. As stretching occurs, the connections and denser network allow the BO/NBO content to mutate earlier and faster, and it thus has a higher brittleness. By contrast, the-high-cooling rate samples have more NBO and small Si-O-Si angles, and the loose network hinders force transfer and requires more energy to fracture, which delays the onset of the BO/NBO content mutation. Therefore, the ductility is greater than that of the low-cooling-rate samples, and they have less brittleness. As a result, MRO should be the most intuitive response unit during the DTB transition and the basic unit that is most directly affected by the stress. As such, the difference in the content of BO and NBO in the glass caused by different cooling rates is an important factor affecting the brittleness. Furthermore, in Figure 7, the 0.25 K/ps sample behaves quite differently from the 1 K/ps sample. Comparing the structural difference between the two before stretching, we can see a significant difference in QnAl, whereas the differences in QnSi and NBO are relatively weak, which can also be seen in Figure 8c,d. Although the system studied in this paper does not have a high Al content, we can still see that Al tetrahedra are more ductile in the network and that QnAl plays a more important role in influencing glass brittleness, and other middle-range structural operations cannot be ignored.

B. Influence of voids

In addition to the different BO and NBO contents of the samples when unstretched, the presence of voids is equally important. When we study the variation in bond length and BO/NBO with stretching, it can be found that they do not always vary linearly according to the magnitude of the cooling rate and with frequently occasional curve fluctuations, so that each curve has its own unique phenomenon (e.g., the anomalous concavity and sudden increase and decrease in the curves of the different cooling rates that are often seen in Figure 7). If there were only one mechanism affecting embrittlement, the curves would not have so many unique features, but they would vary linearly according to the content of the middle-range structure. Therefore, there must be a second mechanism affecting the brittleness. Using the inverse method, we backtracked all different microstructures of glass due to the cooling rates in Section 3.1. It seems that only the size and distribution of voids can plausibly explain these phenomena, which stems from the strong correlation between the voids and the modifier ions in NBO. And in Figure 2b, it appears that the different cooling rates affect the volume of the voids more so than the structure.

**Figure 9:**
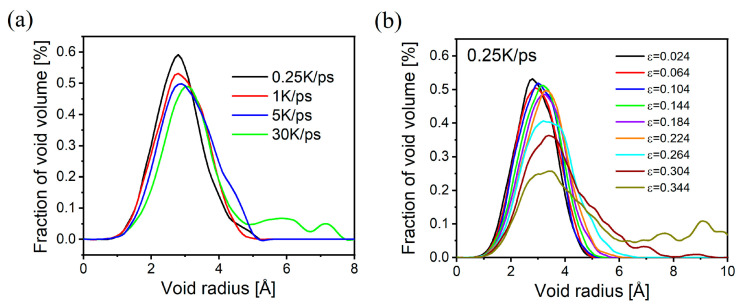
Void distribution for samples with different cooling rates (**a**) and for different strains under stretching of 0.25 K/ps samples (**b**).

In Figure 9, we use the Voronoi-based void analysis method [49] to analyze the void distribution of the glass samples before stretching and at the stage when the brittle/tough transition occurs during stretching. In Figure 9a, the radius of the voids in the glass gradually increases with the increase in the cooling rate, and the distribution becomes wider. This is harmonized with the trend of the glass structure obtained in the previous section. Interestingly, in Figure 9b, the void distribution similarly shows a shift toward larger voids as the tensile strain increases, which is in agreement with the results of other previous studies on sodium silica glasses. And the brittle/tough transition stage exhibits the same pattern regardless of the cooling rate (variations in void distribution during stretching of samples with other cooling rates are in the Supplementary Materials). This indicates that in the process of stress increase, larger voids can provide better ion migration and atomic rearrangement to dissipate stress. Therefore, when the stable structures generated under different cooling rates are stretched, they have different stress dissipation abilities due to the differences in void size and distribution. This allows the BO/NBO to undergo structural reorganization and lead to sudden changes at different levels of strain, resulting in the brittle/ductile transition. And due to the nonuniformity of void distribution, the curves in Figure 7 and Figure 8 undergo occasional fluctuations during stretching.

The effect of voids on tensile has already been found before, and multi-void defects reduce the brittleness and tensile strength of the material but elevate the fracture strain [37,50,51,52]. A similar point is made by Griffiths’ theory of microcracks [53] and the void volume theory proposed by Cohen and Turnbull [54]. However, based on the aforementioned research findings, the more specific role of voids in aluminosilicate glass is the creation of an environment that allows for differential changes in the concentrations of BO and NBO during tensile deformation. This subsequently affects the mode of structural reorganization in the intermediate range structure surrounding the voids during tensile deformation, ultimately impacting the mechanical properties of the glass.

Moreover, in addition to BO/NBO, the ring statistics of glass in tension, which have been found to influence the mechanical properties, can also be explained by the impact of voids on mid-range structures [55,56,57], because the formation of the ring is a manifestation of the BO connectivity network itself. Although only one system has been studied in this paper, i.e., aluminum-silica glass, we believe that the impact of voids on the middle-range structure of the glass is also reflected in other glasses, like in quartz glass, and changing the distribution of QnSi can make a difference in the properties of the glass. Similarly, the theoretical model of the shear transition zone (STZ), proposed by Argon with metallic glasses [58], likewise suggests that voids play a greater role in the plastic flow of clusters of atoms than in the flow of individual atoms. The crucial role of voids in the fracture and reorganization of mesoscale structures during stretching is of great significance in reducing the brittleness and enhancing the ductility of various glasses.

## 4. Conclusions

We studied the potential mechanisms that lead to different brittleness levels of aluminosilicate glass under different cooling rates in this paper. The key role of the abrupt change in the medium-range structure (BO and NBO) content with respect to the occurrence of brittle/tough transition and fracture transition of the material was found. Therefore, the different BO and NBO contents and the diverse void volumes that the glasses with dissimilar cooling rates have both affect the brittleness of the glasses. The former results in different impediments to force transfer by affecting the connectivity of the glass, and the latter leads to different strategies of change in BO and NBO content with respect to increasing strain by affecting the environment of structural rearrangement during stretching, finally causing the glass to yield and fracture at different strains, thus giving rise to different brittleness levels of the glass. At the same time, we identify the main structure of the aluminum-silicate glass affected by voids when subjected to forces, which includes BO and NBO.

In addition, the more ductile nature of aluminum tetrahedra versus silicon tetrahedra likewise plays an important role in aluminosilicate glasses that cannot be ignored. In conclusion, we believe that this study is not limited to aluminosilicate glasses, and the investigation of the location of sudden changes in the mid-range structural content during stretching should be of research value in the study of the mechanical properties of the other types of glasses as well. The results of this paper are also relevant to improving the understanding of the glass brittle/toughness transition and can facilitate the development of tougher glass products.

## Data Availability

Data are contained within the article and Supplementary Materials.

## Supplementary Materials

The following supporting information can be downloaded at: https://www.mdpi.com/article/10.3390/ma17071595/s1. Table S1: Charge for different species. Table S2: Short-range interaction parameters. Figure S1: Void distribution of samples with cooling rates of 1 K/ps (a), 5 K/ps (b), and 30 K/ps (c), stretched to different strains.
