# Supplementary material for: Structural Causes of Brittleness Changes in Aluminosilicate Glasses with Different Cooling Rates
Source: Materials (Basel). 2024 Mar 31;17(7):1595. doi: 10.3390/ma17071595 (PMC11012692; doi:10.3390/ma17071595)
Supplement: Supplementary file 1 [file materials-17-01595-s001.zip › materials-2913454-supplementary.pdf]

Supplementary Materials

S1. Atomic potential information during simulation

The atomic potential developed by Sundararaman et al. is used and takes the form of:

$$V^{buck}(r_{\alpha\beta}) = A_{\alpha\beta}\exp(-B_{\alpha\beta}r_{\alpha\beta}) - \frac{C_{\alpha\beta}}{r_{\alpha\beta}^{6}} + \frac{D_{\alpha\beta}}{r_{\alpha\beta}^{24}} + V^{w}(r_{\alpha\beta})$$

Where:

$$V^{w}(r_{\alpha\beta}) = q_{\alpha}q_{\beta}\left[\frac{1}{r_{\alpha\beta}} - \frac{1}{r_{cut}^{w}} + \frac{(r_{\alpha\beta} - r_{cut}^{w})}{(r_{cut}^{w})^{2}}\right]$$

$r_{\alpha\beta}$ is the distance between atoms α and β, $A_{\alpha\beta}$, $B_{\alpha\beta}$ and $C_{\alpha\beta}$ are energy parameters for the pair ij describing repulsive and dispersive forces. The repulsive term $\frac{D_{\alpha\beta}}{r_{\alpha\beta}^{24}}$ is added to avoid the divergence of the potential due to the van der waals term at very small distances. $V^{w}(r_{\alpha\beta})$ is the Wolf truncation method to evaluated the long-range Coulomb interactions, r_{cut}^{w} is a cutoff distance. And the short-range interactions are truncated at 8 Å and V^{w} at 10 Å.

The charge of the oxygen atoms for the SHIK potentials depends on composition in order to maintain charge neutrality of the system when the sodium concentration is varied:

$$q_{O} = \frac{(1-y_{Na}-y_{Al})q_{Si}+2y_{Na}q_{Na}+2y_{Al}q_{Al}}{y_{Na}-y_{Al}-2};$$

where y_{Na}, y_{Al} is the Na2O and Al2O3 mole concentration, respectively.

Table S1 lists the calculated charge, and all the parameters used in the potential function list in the Table S2, and the charge of oxygen is calculated from the glass composition 25Na2O-5Al2O3-70SiO2 (mol. %):

Table S1. Charge for different species.

Species	Si	Al	Na	O
Charge (e)	1.7755	1.6334	0.6018	-0.9484

Table S2. Short-range interaction parameters.

	$A_{\alpha\beta}$(eV)	$B_{\alpha\beta}$ (Å^{-1})	$C_{\alpha\beta}$ (eV·Å^6)	$D_{\alpha\beta}$ (eV·Å^{24})
O-O	1120.5	2.8927	26.132	16800
O-Si	23108	5.0979	139.70	66
Si- Si	2798.0	4.4073	0.0	3423204
O-Na	1127566	6.8986	40.562	16800
Si-Na	495653	5.4151	0.0	16800
Na-Na	1476.9	3.4075	0.0	16800
O-Al	21740	5.3054	65.815	66
Al-Al	1799.1	3.6778	100.0	16800

S2. Stress-strain curve calculation method

To calculate the stress–strain curve we increased the dimension of the box in one direction linearly in time,

$$L(t) = L(0)[1 + \varepsilon(t)]$$

The strain is then given by:

$$\varepsilon(t) = \acute{\varepsilon}t$$

where $\acute{\varepsilon}$ is the strain rate.

The stress of the tensile process was plotted by integrating the total stress experienced by all atoms in the y-direction under varying strains The stress tensor is obtained from the usual expression:

$$\sigma = \frac{1}{V}\sum_{i=1}^{N}[m_i v_i \otimes \ vi + r_i \ \otimes \ f_i]$$

where V and N are the volume and the total number of atoms of the simulation box, respectively, while m_i is the mass of atom i, and v_i, r_i and f_i are the velocity, position and force vector of atom i, respectively.

S3. Voids distribution

Figure S1 shows the void distribution (brittle/tough transition phase) for samples stretched to different strains at different rates in sequence. All three samples had a tendency for the voids to become larger as they were stretched, and the curve shifted to the right overall.

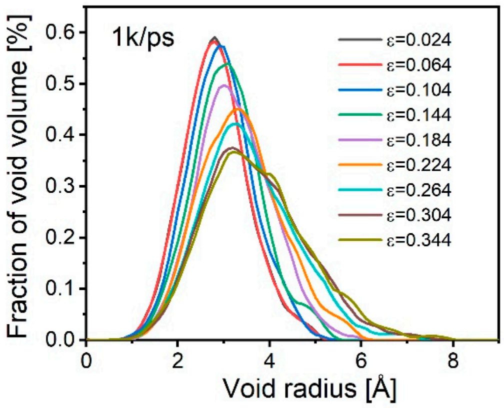

(a)

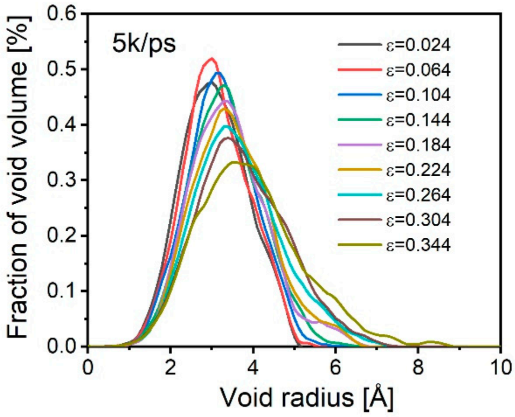

(b)

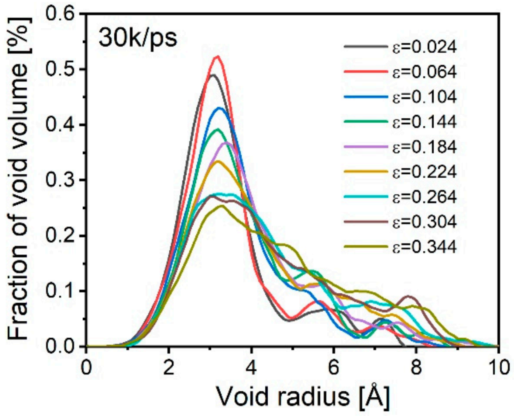

(c)

Figure S1. Void distribution of samples with cooling rates of 1 K/ps (a), 5 K/ps (b), and 30 K/ps (c), stretched to different strains
